# Draft genome sequence of *Thermoactinomyces* sp. strain AS95 isolated from a Sebkha in Thamelaht, Algeria

**DOI:** 10.1186/s40793-016-0186-2

**Published:** 2016-09-09

**Authors:** Oliver K. I. Bezuidt, Mohamed A. Gomri, Rian Pierneef, Marc W. Van Goethem, Karima Kharroub, Don A. Cowan, Thulani P. Makhalanyane

**Affiliations:** 1 Centre for Microbial Ecology and Genomics, Department of Genetics, University of Pretoria, Natural Sciences 2 Building, Office 3-14, Lynnwood Road, Pretoria, 0028 South Africa; 2 Biotechnology Platform, Agricultural Research Council, Pretoria, South Africa; 3 Equipe Métabolites des Extrêmophiles, Laboratoire de Recherche Biotechnologie et Qualité des Aliments, INATAA, Université Frères Mentouri Constantine, Constantine, Algérie; 4 Centre for Bioinformatics and Computational Biology, Department of Biochemistry, University of Pretoria, Pretoria, 0028 South Africa

**Keywords:** *Thermoactinomyces* sp. strain AS95, Genome, Thermophilic, Proteolytic activity, Taxonomo-genomics

## Abstract

The members of the genus *Thermoactinomyces* are known for their protein degradative capacities. *Thermoactinomyces* sp. strain AS95 is a Gram-positive filamentous bacterium, isolated from moderately saline water in the Thamelaht region of Algeria. This isolate is a thermophilic aerobic bacterium with the capacity to produce extracellular proteolytic enzymes. This strain exhibits up to 99 % similarity with members of the genus *Thermoactinomyces,* based on 16S rRNA gene sequence similarity. Here we report on the phenotypic features of *Thermoactinomyces* sp. strain AS95 together with the draft genome sequence and its annotation. The genome of this strain is 2,558,690 bp in length (one chromosome, but no plasmid) with an average G + C content of 47.95 %, and contains 2550 protein-coding and 60 RNA genes together with 64 ORFs annotated as proteases.

## Introduction

Modern metagenomic approaches have provided insights on the evolution and functional capacity of microbial communities resistant to classical culture-based methods [1]. However, these classical techniques remain crucial for understanding the molecular adaptations of microbial guilds, especially those with potential biotechnological applications [2, 3]. Consequently, efforts to isolate novel taxa, particularly from environmentally extreme habitats remain widespread [4, 5].

The genus *Thermoactinomyces* is a member of the family *Thermoactinomycetaceae**.* The first known representative from this genus (*Thermoactinomyces vulgaris*) was isolated from decaying straw and manure [6]. Since then, a number of isolates, from a wide array of extreme habitats [7–10] have been validly described. Currently, this genus comprises ten validly published species, and a few of these are; *Thermoactinomyces vulgaris* [6], *Thermoactinomyces intermedius* [11], *Thermoactinomyces daqus* [7] and *Thermoactinomyces guangxiensis* [8]. These species are all Gram-positive, aerobic, non-acid-fast, chemoorganotrophic, filamentous and thermophilic bacteria.

Here, we report the draft genome sequence of *Thermoactinomyces* sp. strain AS95, which was isolated from a sebkha (endorheic salt pan) in the Thamelaht region ofAlgeria. We present a summary of the classification and set of phenotypic features for *Thermoactinomyces* sp. strain AS95 together with the description of the non-contiguous genome sequence and its annotation with particular reference to ORFs encoding proteolytic enzymes.

## Organism information

### Classification and features

**Fig. 1:**
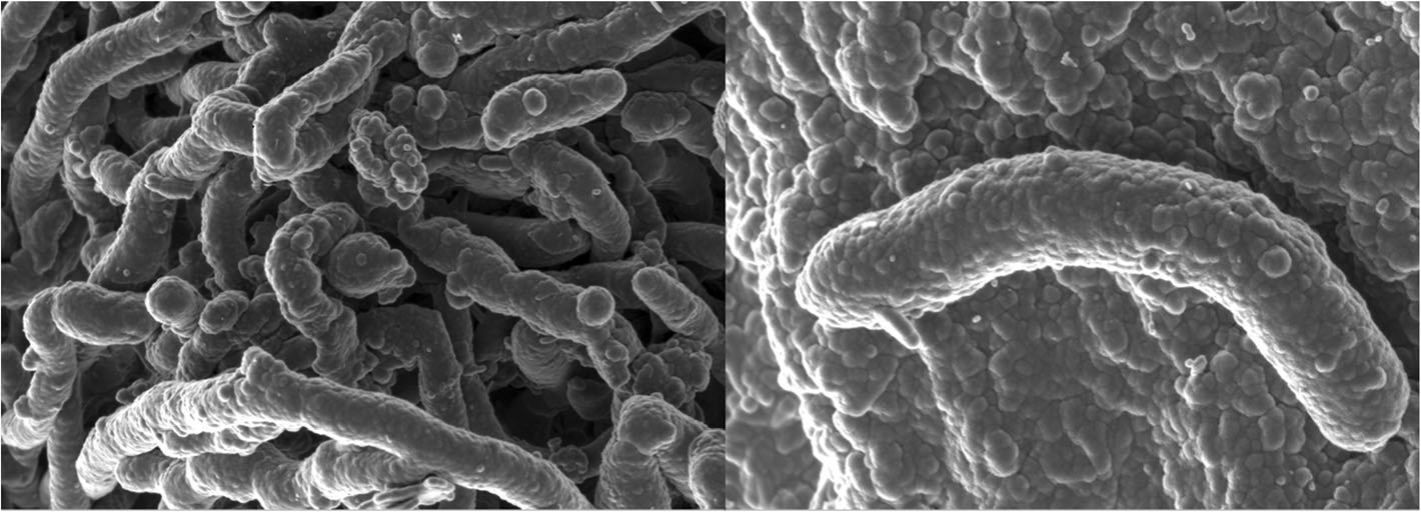
Scanning electron microscopy of *Thermoactinomyces* sp. strain AS95 using a Cryo-SEM (JEOL)

**Fig. 2:**
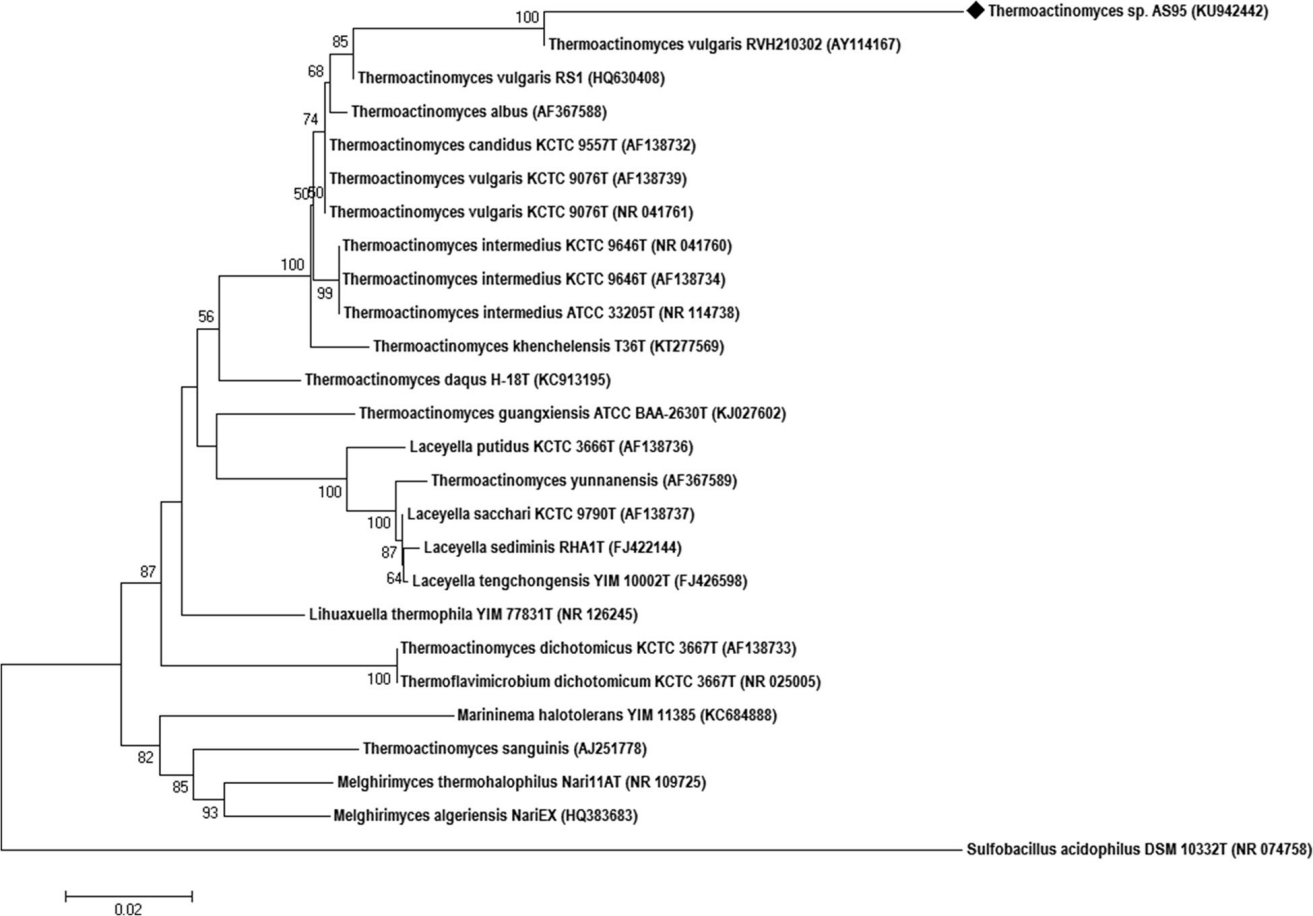
Phylogenetic tree based on 16S rRNA gene sequences showing the relationship between strain AS95 (1435 bp) and strains of related genera of the family *Thermoactinomycetaceae*. The strains and their corresponding Genbank accession numbers are shown following the organism name and indicated in parentheses. The phylogenetic tree was made using the neighbor-joining method with maximum composite likelihood model implemented in MEGA 7. The tree includes the 16S rRNA gene sequence of *Sulfobacillus acidophilus* DSM 10332^T^ as outgroup. Bootstrap consensus trees were inferred from 1000 replicates, only bootstrap values >50 % are indicated. The scale bar represents 0.02 nucleotide changes per position. (♦) indicates the isolate assessed in the current study, *Thermoactinomyces* sp. strain AS95

*Thermoactinomyces* strain AS95 was isolated from a sebkha water sample collected in June 2013 from the Thamelaht region ofAlgeria (Table 1). This isolate is a Gram-positive, aerobic, thermophilic, filamentous bacterium (Fig. 1) belonging to the order *Bacillales*. Based on the 16S rRNA gene sequence similarity searches by BLASTN against the NCBI-NT database, strain AS95 showed 97–99 % sequence similarity to members of the genus *Thermoactinomyces*. A 16S rRNA gene-based phylogenetic tree of *Thermoactinomyces* sp. strain AS95 was constructed (Fig. 2), based on neighbor-joining and maximum composite likelihood models with 1000 bootstrap replications using MEGA 7 [12]. The *Thermoactinomyces* sp. strain AS95 (KU942442) 16S rRNA gene sequence exhibited high identity (99 %) with *Thermoactinomyces vulgaris* RVH210302 (AY114167), the closest validly published *Thermoactinomyces* species.

**Table 1:** Classification and general features of *Thermoactinomyces* sp. strain AS95

MIGS ID	Property	Term	Evidence code^a^
	Classification	Domain: *Bacteria*	TAS [20]
		Phylum: *Firmicutes*	TAS [21–23]
		Class: *Bacilli*	TAS [24, 25]
		Order: *Bacillales*	TAS [26, 27]
		Family: *Thermoactinomycetaceae*	TAS [25, 28]
		Genus: *Thermoactinomyces*	TAS [6]
		Species: *Thermoactinomyces* sp.	IDA
		Strain: AS95	IDA
	Gram stain	Positive	IDA
	Cell shape	Filamentous	IDA
	Motility	Non-motile	IDA
	Sporulation	Endospores on unbranched sporophores	IDA
	Temperature range	40–65 °C (Thermophilic)	IDA
	Optimum temperature	55 °C	IDA
	pH range; Optimum	5.6–8.6; 7.2	IDA
	Carbon source	Peptides	IDA
GS-6	Habitat	Saline water	IDA
MIGS-6.3	Salinity	5.0 % total salt (w/v)	IDA
MIGS-22	Oxygen requirement	Aerobic	IDA
MIGS-15	Biotic relationship	Free-living	IDA
MIGS-14	Pathogenicity	Non-pathogen	IDA
MIGS-4	Geographic location	Thamelaht,, Algeria	IDA
MIGS-5	Sample collection time	20 June 2013	IDA
MIGS-4.1	Latitude	36°32'18.29"N	IDA
MIGS-4.2	Longitude	5°11'48.89"E	IDA
MIGS-4.4	Altitude	890 m above sea level	IDA

^a^Evidence codes – IDA: Inferred from Direct Assay; TAS: Traceable Author Statement (i.e. a direct report exists in the literature). These evidence codes are from the Gene Ontology Project [29]. If the evidence is IDA, then the property was directly observed for a live isolate by one of the authors or an expert mentioned in the acknowledgements

The strain was cultivated on *Thermus* medium agar containing 2.0 g NaCl, 4.0 g yeast extract, 8.0 g peptone and 30.0 g agar per liter of distilled water. The bacterium grew optimally at 55 °C, with a broad temperature growth range of between 40 and 65 °C (Table 1). The strain grew in liquid media at pH values from 5.6 to 8.6, but optimal growth occurred at a pH of 7.2. Morphologically, the isolate forms white colonies and abundant aerial mycelia with the appearance of well-developed, branched and septate substrate mycelia. The micromorphology of the cells was examined using scanning electron microscopy (Fig. 1). The predominant menaquinone was MK-7. Major fatty acids included iso-C15:0, and significant amounts of iso-C17:0 were also present.

## Genome sequencing information

### Genome project history

A high-quality draft genome sequence is deposited at DDBJ/EMBL/GenBank under the accession LSVF00000000 and consists of 11 scaffolds of 11 contigs. A summary of the project information and its association with MIGS version 2.0 compliance are shown in Table 2 [13].

**Table 2:** Project information

MIGS ID	Property	Term
MIGS-31	Finishing quality	High-quality draft
MIGS-28	Libraries used	One paired-end 300 bp library
MIGS-29	Sequencing platforms	MiSeq-Illumina
MIGS-31.2	Fold coverage	40.0×
MIGS-30	Assemblers	SPAdes 3.5.0
MIGS-32	Gene calling method	NCBI Prokaryotic Genome, Annotation Pipeline
	Genbank ID	LSVF00000000
	Genbank Date of Release	April 04, 2016
	BIOPROJECT	PRJNA312744
	GOLD ID	Gs0118400
MIGS-13	Project relevance	Biotechnological, Environmental

### Growth conditions and genomic DNA preparation

*Thermoactinomyces* sp. strain AS95 was grown aerobically on *Thermus* medium agar (pH 7.2) at 55 °C for 24 h. Genomic DNA was extracted using a modification of a previously described protocol [14]. The quantity and quality of the genomic DNA was measured using a NanoDrop Spectrophotometer and a Qubit™ Fluorometer (Thermo Fisher Scientific Inc.).

### Genome sequencing and assembly

Genomic DNA samples of *Thermoactinomyces* sp. strain AS95 were sequenced at MR DNA (Shallowater, TX, USA). Genome sequencing was performed on a MiSeq (Illumina, Inc.) generating 2 x 300 bp paired-end libraries. The sequencing run produced a total of 5,085,250 reads, with a mean length of 265.58 bp. The raw paired-end sequences were subjected to the fastxtools software [15] for quality trimming using a phred quality score ≥ 20. After trimming, a total of 3,013,639 reads with a mean length of 171.11 bp were assembled using SPAdes, version 3.5.0 [16]. The final assembly resulted in a total of 11 scaffolds, which generated a genome size of 2.56 Mb.

### Genome annotation

Genome annotation was carried out on the RAST server [17] and using the NCBI Prokaryotic Genome Annotation Pipeline tools [18]. This Whole Genome Shotgun sequence project has been deposited at DDBJ/EMBL/GenBank under accession LSVF00000000. The version described in this paper is version LSVF00000000.

## Genome properties

The genome is composed of 2,558,690 nucleotides with 47.95 % G + C content (Table 3) and comprised 11 scaffolds of 11 contigs. The genome contains a total of 2649 genes, 2550 of which were protein coding, 39 pseudogenes and 60 RNA coding genes. The majority of protein-coding genes (75.45 %) were assigned a putative function while the remaining genes were annotated as hypothetical. The distribution of genes in COGs functional categories is presented in Table 4.

**Table 3:** Genome statistics of the *Thermoactinomyces* sp. strain AS95

Attribute	Value	% of total^a^
Genome size (bp)	2,558,690	100.00
DNA coding region (bp)	2,214,681	86.56
DNA G + C (bp)	1,226,817	47.95
DNA scaffolds	11	
Total genes	2,649	100.00
Protein coding genes	2,550	96.26
RNA genes	60	2.26
Pseudo genes	39	1.47
Genes in internal clusters	ND	ND
Genes with function prediction	1,296	50.82
Genes with Pfam domains	2,001	78.47
Genes assigned to COGs	1,924	75.45
Genes with signal peptides	164	6.43
Genes with transmembrane helices	655	25.69
CRISPR repeats	2	ND

^a^The total is based on either the size of the genome in base pairs or the total number of protein coding genes in the annotated genome. ND: Not determined

**Table 4:** Number of genes associated with general COG functional categories

Code	Value	% of total^a^	Description
J	154	9.96	Translation, ribosomal structure and biogenesis
A	0	0.00	RNA processing and modification
K	145	5.68	Transcription
L	100	3.92	Replication, recombination and repair
B	0	0.00	Chromatin structure and dynamics
D	27	1.05	Cell cycle control, mitosis and meiosis
V	32	1.25	Defense mechanisms
T	71	2.78	Signal transduction mechanisms
M	99	3.88	Cell wall/membrane biogenesis
N	8	0.31	Cell motility
Z	0	0.03	Cytoskeleton
U	33	1.29	Intracellular trafficking and secretion
O	85	3.33	Posttranslational modification, protein turnover, chaperones
C	135	5.29	Energy production and conversion
G	122	4.78	Carbohydrate transport and metabolism
E	213	8.35	Amino acid transport and metabolism
F	70	2.74	Nucleotide transport and metabolism
H	108	4.23	Coenzyme transport and metabolism
I	109	4.27	Lipid transport and metabolism
P	101	3.96	Inorganic ion transport and metabolism
Q	53	2.07	Secondary metabolites biosynthesis, transport and catabolism
R	249	9.76	General function prediction only
S	196	7.68	Function unknown
-	626	24.54	Not in COGs

^a^The total is based on the total number of protein coding genes in the annotated genome

A blastp comparison was conducted against the MEROPS database. A total of 64 protein-coding genes (2.4 %) were predicted to share homology with various categories of proteases (Table 5). Of these predictions indicated that 36 were putatively secreted in a classical pathway (SignalP), whereas the other 28 were secreted in a non-classical pathway (SecretomeP). Only 2 of the 64 protein-coding genes share sequence similarities with proteases of the *Thermoactinomyces vulgaris* and sp. E79 families of peptidases in the MEROPS database.

**Table 5:** The four major types of proteases predicted in *Thermoactinomyces* sp. strain AS95

Type	Classical (SignalP)	Non-classical (SecretomeP)
Cysteine	6	3
Metallo	18	12
Serine	11	10
Threonine	0	2

## Conclusions

This study describes the draft genome sequence of *Thermoactinomyces* sp. strain AS95, which is associated with a high level of extracellular proteolytic activities. To date, only a few metabolic pathways involved in protein degradation have been characterized for the genus *Thermoactinomyces* [19]. The genome sequence and characteristics of strain AS95 will provide new insights into the mechanisms of protein degradation in the genus *Thermoactinomycetes,* and towards establishing a comprehensive genomic catalog of the metabolic diversity of the genus *Thermoactinomyces*.
